# Genome-Wide Analysis of the *Rhododendron* AP2/ERF Gene Family: Identification and Expression Profiles in Response to Cold, Salt and Drought Stress

**DOI:** 10.3390/plants12050994

**Published:** 2023-02-22

**Authors:** Zhenhao Guo, Lisi He, Xiaobo Sun, Chang Li, Jiale Su, Huimin Zhou, Xiaoqing Liu

**Affiliations:** 1 Institute of Leisure Agriculture, Jiangsu Academy of Agricultural Sciences, Nanjing 210014, China; 2 Jiangsu Key Laboratory for Horticultural Crop Genetic Improvement, Nanjing 210014, China

**Keywords:** *Rhododendron*, transcription factor, genome-wide, abiotic stress, gene duplication, gene expression

## Abstract

The AP2/ERF gene family is one of the most conserved and important transcription factor families mainly occurring in plants with various functions in regulating plant biological and physiological processes. However, little comprehensive research has been conducted on the AP2/ERF gene family in *Rhododendron* (specifically, *Rhododendron simsii*), an important ornamental plant. The existing whole-genome sequence of *Rhododendron* provided data to investigate the AP2/ERF genes in *Rhododendron* on a genome-wide scale. A total of 120 *Rhododendron* AP2/ERF genes were identified. The phylogenetic analysis showed that RsAP2 genes were classified into five main subfamilies, AP2, ERF, DREB, RAV and soloist. Cis-acting elements involving plant growth regulators, response to abiotic stress and MYB binding sites were detected in the upstream sequences of RsAP2 genes. A heatmap of RsAP2 gene expression levels showed that these genes had different expression patterns in the five developmental stages of *Rhododendron* flowers. Twenty RsAP2 genes were selected for quantitative RT-PCR experiments to clarify the expression level changes under cold, salt and drought stress treatments, and the results showed that most of the RsAP2 genes responded to these abiotic stresses. This study generated comprehensive information on the RsAP2 gene family and provides a theoretical basis for future genetic improvement.

## 1. Introduction

As a widely distributed ornamental genus around the world, it is difficult to deny that *Rhododendron* has more dimensions of ornamental diversity than other popular woody horticultural plants. In China, *Rhododendron simsii* is the most widely cultivated *Rhododendron* species in landscaping, and numerous cultivars have been obtained through hybridization [1]. *Rhododendron* flower buds generally undergo a period of dormancy between flower bud differentiation and anthesis [2] to cope with the environmental conditions of autumn and winter. Previous studies have shown that *Rhododendron* flower buds are more sensitive to cold than are leaf buds, leaves and stems, and that the cold-tolerance gap between flower tissues and vegetative tissues is more obvious in plants with strong cold tolerance [3]. The critical low temperature that flower buds can tolerate before damage occurs is regarded as an important trait indicator [4]. Therefore, cold tolerance is one of the most important prerequisites for maintaining ornamental characteristics. In addition to temperature, various abiotic stress factors such as drought and high soil salt concentrations, not only affect the overall health of plants but also cause damage to flower buds [5,6]. Gardeners formerly used cross-breeding to obtain varieties with high abiotic stress tolerance. However, this method was less efficient in terms of time and the resulting varieties were often difficult to combine ornamental traits together with abiotic stresses resistance [4]. It is necessary to understand the molecular mechanism related to flower stress resistance to meet the demand. Recent research on the rose APERALA2/Ethylene Responsive Factor (AP2/ERF) gene family showed that the isolated member gene *RcDREB2B* is repressed under drought stress, while the overexpression of *RcDREB2B* was found to promote sensitivity to salt, Abscisic Acid (ABA) and poly (ethylene glycol) [7]. Enhancing abiotic stress tolerance through molecular breeding can enable the barriers of traditional breeding methods to be overcome and can influence the regional distribution of cultivation [4].

The AP2/ERF superfamily is one of the most conserved and important Transcription Factor (TF) families and is mainly found plants [8]. This TF family has various functions for regulating plant biological and physiological processes, including responses to abiotic stress and contributions to developmental processes and plant morphogenesis [9]. AP2/ERF proteins are recognized with AP2 binding domains, and the APE/ERF family can be divided into five subfamilies: AP2, Dehydration-Responsive Element-Binding (DREB) protein, ERF, ABI3/VP1 (RAV) and soloist, according to the features of the domains [8]. Different structural characteristics determine the differences in function among these subfamilies, while the AP2 subfamily members contribute to the regulation of anthesis and development of flower organs [10,11]. The ERF and DREB subfamily members mainly regulate the response toward abiotic stresses such as cold and salt [12,13], and the RAV subfamily members participate in the regulation of plant hormones such as ethylene and brassinolide [14]. The soloist subfamily function requires more research to clarify.

It has been reported in *Arabidopsis thaliana* that exogenously applied abscisic acid, cold, dehydration and high salinity will induce the expression of genes with cis-acting elements sharing a conserved ‘A/GCCGAC’ core sequence [15,16,17,18]. One of the cis-elements, TACCGACAT, was named the Dehydration-Responsive Element (DRE) and can bind to the TFs named DRE-Binding Protein 1/C-repeat Binding Factor (DREB1/CBF) and DREB2 [19,20]. Overexpression of DREB1/CBF ultimately improves the tolerance to cold, drought and high salinity in *Arabidopsis*, while DREB2 can help counter the stress caused by drought, high salinity and heat shock through a series of expression changes in downstream genes [21,22,23]. AP2/ERF transcription factors have been widely detected in plants, and their functions in flower physiology and development have been studied, including in *Arabidopsis* [24], tomato [25] and maize [26], while their functions in abiotic stress response have also been studied, mainly in model and crop plants such as rice [27] and wheat [28]. However, in ornamental plants, where research on flower development is more important and prominent, there is no comprehensive understanding of the AP2/ERF transcription factor family as there is in food-crop plants. In flowers, an important reproductive organ, there is still a lack of research on the AP2/ERF gene family and the mechanism toward abiotic stress resistance, and the need for obtaining more bioinformatics data. The achievement of the *Rhododendron* whole-genome sequence provides opportunities to comprehensively study the *Rhododendron* AP2/ERF gene family.

In this study, we identified AP2/ERF genes from the *Rhododendron* genome using bioinformatics methods and obtained complete information such as sequence features, classification, and chromosomal and promoter locations. We generated more understanding of the RsAP2 genes’ function through determining the expression patterns of the AP2/ERF genes in different *Rhododendron* flower developmental stages and the expression levels in closed buds under different abiotic stresses, including cold, salt and drought. This study provides a theoretical basis for the further validation of RsAP2 gene function and protein interaction.

## 2. Results

### 2.1. Identification and Analysis of RsAP2 Genes

A total of 120 AP2 genes were obtained through Hidden Markov Model (HMM) searches with the *Rhododendron*-specific AP2 model, and their AP2 domains were confirmed by the Simole Modular Architecture Research Tool (SMART), the National Center for Biotechnology Information Conserved Domains Database (NCBI CDD) and Pfam. The sequences were named according to their chromosomal location. Gene characteristics, including gene names, IDs, subfamilies, length of proteins, molecular weight of proteins (MW), isoelectric points (pI), chromosomal locations and main subcellular localizations are displayed in Table S1. Among all of the RsAP2 proteins, RsAP2-59 was identified as the largest protein with 906 aa and 100.7 kDa, while RsAP2-16 was the smallest, with 129 aa and 13.6 kDa in protein length and MW, respectively. The results of subcellular localization prediction showed that RsAP2 proteins were mainly located in the nucleus, followed by the chloroplast.

### 2.2. Phylogenetic Analysis and Classification of RsAP2 Genes

**Figure 1:**
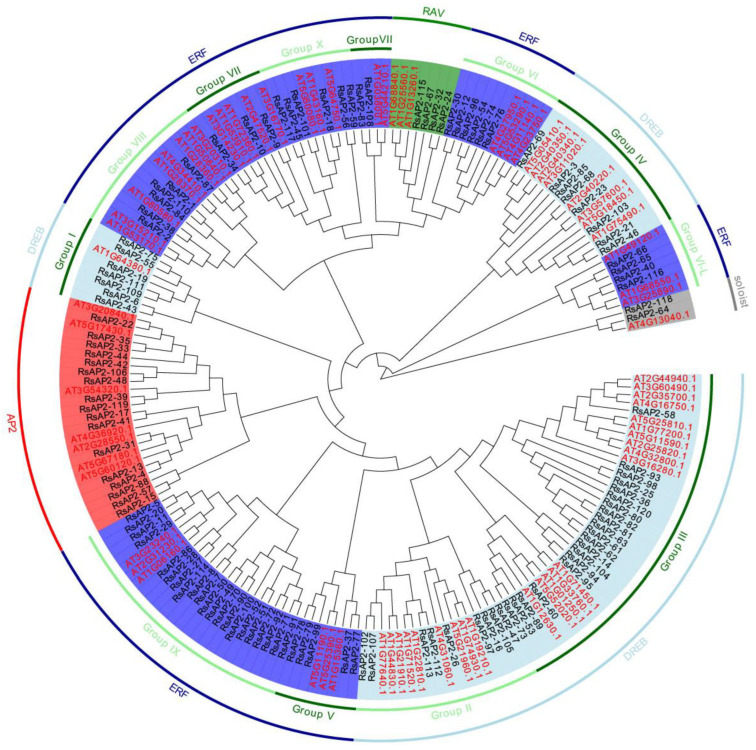
Phylogenetic tree of AP2 genes from *Rhododendron* and *Arabidopsis*. The identifiers colored in black are RsAP2 genes and the AP2 genes from *Arabidopsis* are colored in red. Subfamilies of AP2, DREB, ERF, RAV and soloist are distinguished by backgrounds shown in red, light blue, dark blue, green and gray, respectively.

A phylogenetic tree of AP2 genes in *Rhododendron* and *Arabidopsis* was constructed to analyze the evolutionary relationship. A total of 195 AP2 genes were classified into 5 subfamilies, and the DREB and ERF subfamilies were further divided into 11 subgroups, as shown in Figure 1. Seventeen RsAP2 genes were classified into the AP2 subfamily with two tandem-duplicated AP2 domains. The DREB and ERF subfamilies both have only one AP2 domain. Forty-four RsAP2 genes with the 14th and 19th amino acids of the domain being valine and glutamic acid, respectively, were confirmed to be DREB subfamily members. Fifty-three RsAP2 genes with the 14th and 19th amino acids of the domain being alanine and aspartic acid, respectively, were confirmed to be ERF subfamily members. Four RsAP2 genes with an AP2 domain at the C-terminus and a B3 domain at the N-terminus were recognized as RAV subfamily members. The remaining two RsAP2 genes with an AP2 domain but with large differences in gene structure from other subfamily members were classified into the soloist subfamily. As shown in the figure, AP2 genes with the same structural features were accurately clustered into a certain subfamily, which indicated that the AP2 gene family has high homology across different species.

### 2.3. RsAP2 Protein Motifs and Gene Structure Analysis

**Figure 2:**
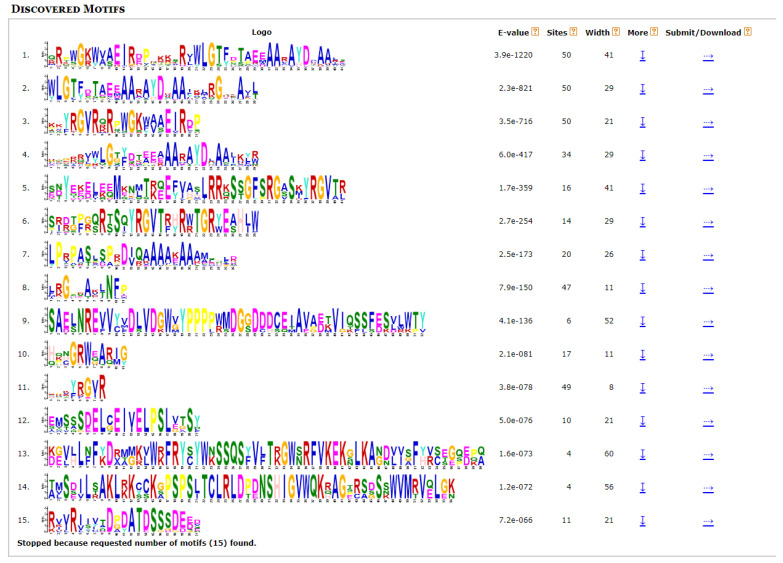
Fifteen main conserved motifs of RsAP2 genes.

**Figure 3:**
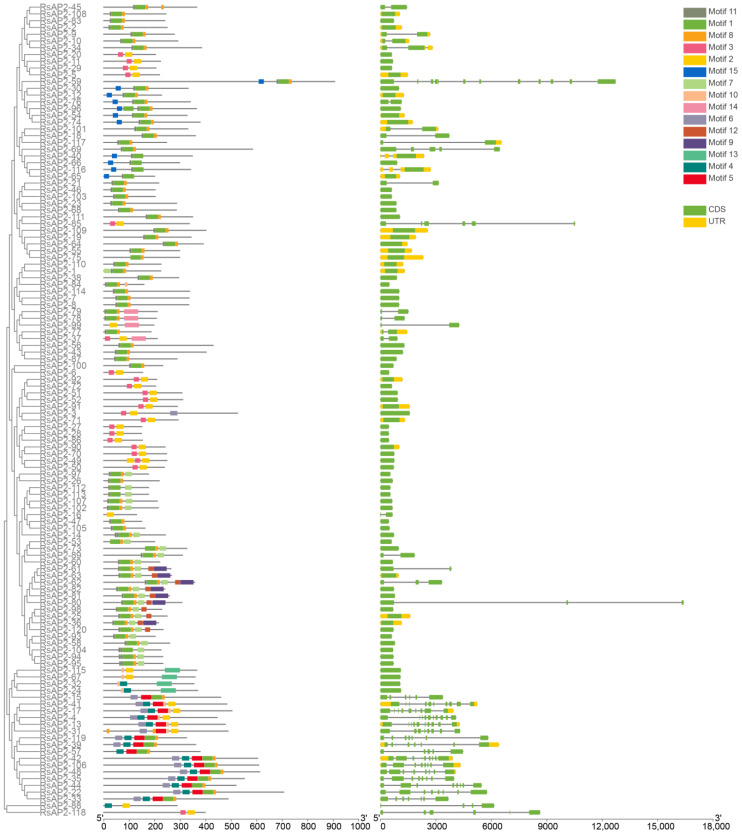
Chromosomal distribution and gene duplication analysis of RsAP2 genes.

Fifteen conserved motifs of RsAP2 proteins were identified with Multiple EM for Motif Elicitation (MEME) software. These motifs were composed of 8–60 amino acids as shown in Figure 2. The types and distribution of motifs were different in RsAP2 genes, but several combined patterns of motifs, such as motif 11-1-8 and motif 3-2, appear rather frequently. Gene structures of introns and exons were acquired from alignment of genome sequence data. Among the RsAP2 genes, 79 genes had no introns, 17 genes had 1 intron and 24 genes had 2 or more introns. To intuitively compare the features of the RsAP2 genes, Figure 3 was constructed by using a phylogenetic tree, the distribution of motifs and the gene structure of RsAP2 genes.

### 2.4. Chromosome Location and Gene Duplication of RsAP2 Genes

**Figure 4:**
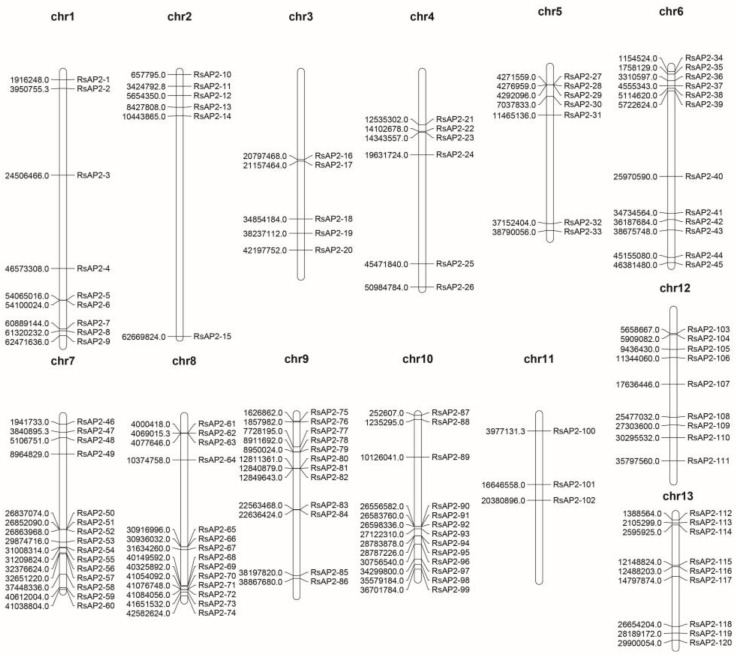
Chromosomal distribution and location of RsAP2.

The 120 RsAP2 genes were distributed on the thirteen chromosomes unevenly, with the chromosome 7 containing fifteen of the RsAP2 genes and the chromosome 11 contained only three RsAP2 genes, which were the largest and smallest numbers, respectively (Figure 4).

**Figure 5:**
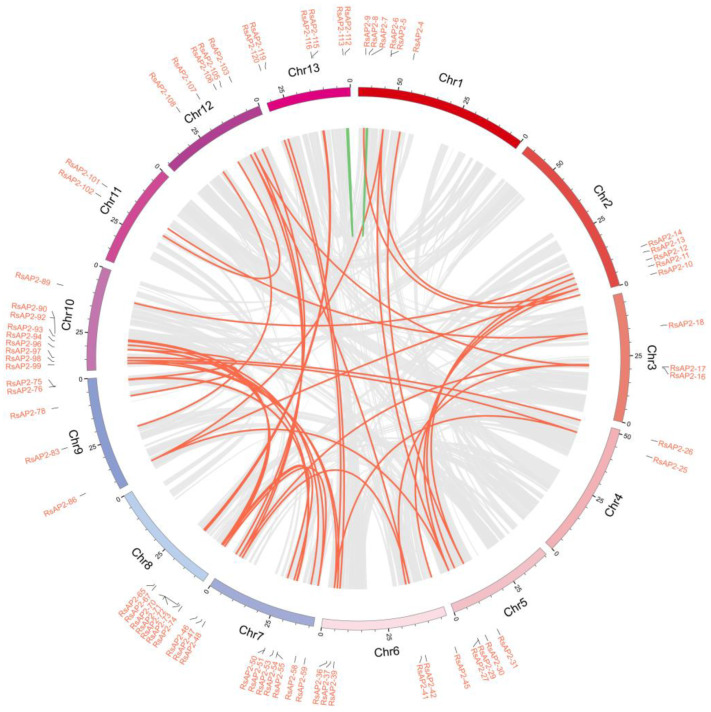
Synteny analysis and chromosomal relationships of *Rhododendron* RsAP2 genes. Red lines indicate duplicated RsAP2 gene pairs between different chromosomals, green lines indicate duplicated RsAP2 gene pairs in the same chromosomal and gray lines indicate all synteny blocks in the *Rhododendron* genome.

The evolution of genomes and genetic systems is considered to be driven by gene duplications and could be distinguished as segmental duplication and tandem duplication [29]. A tandem duplication event is described as a chromosomal region within 200 kb containing two or more genes, according to previous research [30]. As shown in Figure 5, thirty RsAP2 genes (RsAP2-5/6, RsAP2-27/28/29, RsAP2-50/51/52, RsAP2-61/62/63, RsAP2-65/66, RsAP2-68/69, RsAP2-70/71/72, RsAP2-78/79, RsAP2-80/81/82, RsAP2-83/84, RsAP2-90/91/92 and RsAP2-94/95) were clustered into twelve tandem duplication event regions on *Rhododendron* chromosomes 1, 5, 7, 8, 9 and 10. The most frequent AP2/ERF gene tandem duplication event occurred on chromosome 8 with four clusters. Fifty segmental duplication events involving seventy-one RsAP2 genes were identified with Multiple Collinearity Scan toolkit X version software (MCScanX). The tandem and segmental duplication contributed greatly to the expansion and evolution process of RsAP2, especially for the latter pattern.

### 2.5. Important Cis-Acting Elements in RsAP2 Promoters

**Figure 6:**
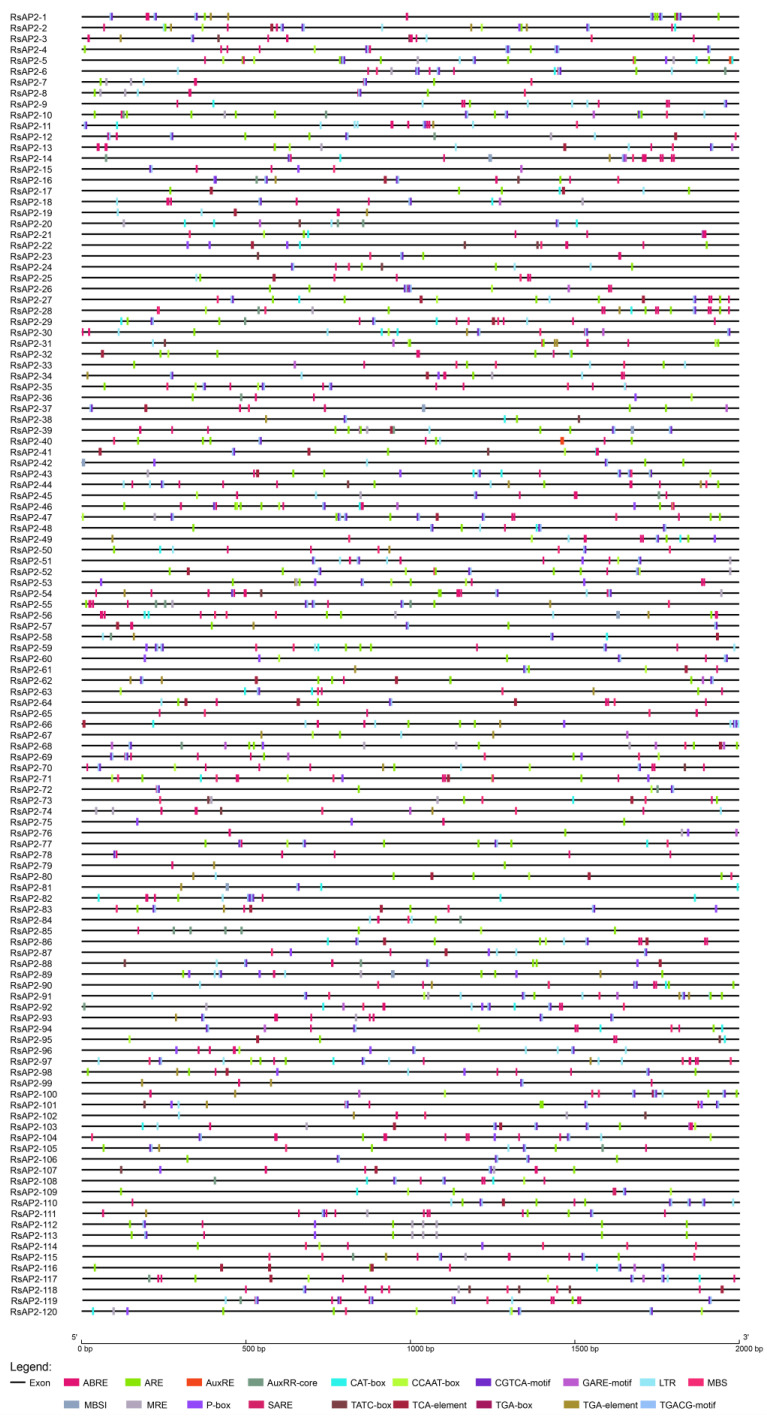
Cis-acting elements in *RsAP2* promoters.

Upstream sequences (2 kb) were extracted according to the chromosomal locations of RsAP2 genes. Cis-acting elements were detected by using the Plant Cis-Acting Regulatory Element (PlantCARE) and the results showed elements with several putative functions. These elements were divided into three main categories according to their putative functions, including plant growth regulation, responses to abiotic stress and MYB binding sites (Figure 6). The cis-acting elements ABRE and LTR, which respond to abscisic acid and are involved in low-temperature responsiveness, respectively, were widely found as promoters of the 106 and 68 RsAP2 genes, respectively. These findings indicate the vital function of RsAP2 in counteracting environmental stress, especially cold stress.

### 2.6. Expression Patterns of RsAP2 Genes in Different Stages of the Flowering Process

**Figure 7:**
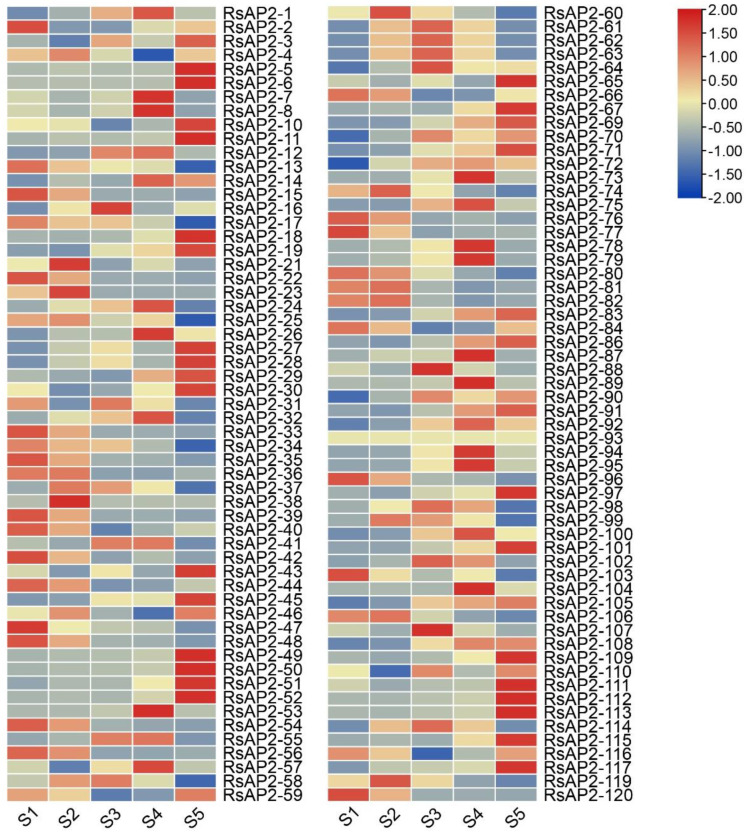
Heatmap of expression levels of RsAP2 genes in different development stages of *Rhododendron* flowers. The FPKM levels are transformed by log2 and labels of S1, S2, S3, S4 and S5 represent closed buds, buds showing color at the top but with the scales still present, initial flowering stage, full flowering stage and last flowering stage, respectively.

The heatmap of 115 RsAP2 genes (with 5 RsAP2 genes eliminated through the results of local Basic Local Alignment Search Tool, BLAST) was generated by the Fragments per Kilobase of Transcript per Million Mapped reads (FPKM) values, transformed by log2 (Figure 7). The RsAP2 genes had different expression patterns across the five development stages of *Rhododendron* flowers. Most of the RsAP2 genes had an especially high expression stage that was distinct from the others. Almost all of the RsAP2 gene clusters with tandem duplication events, such as RsAP2-5/6 and RsAP2-50/51/52, showed similar expression patterns, and they all had higher expression levels in the last flowering stage. Additionally, the cluster of RsAP2-80/81/82 had higher expression levels in the S1 and S2 stages but lower expression levels in other stages.

### 2.7. Interaction Network Analysis of RsAP2 Proteins

**Figure 8:**
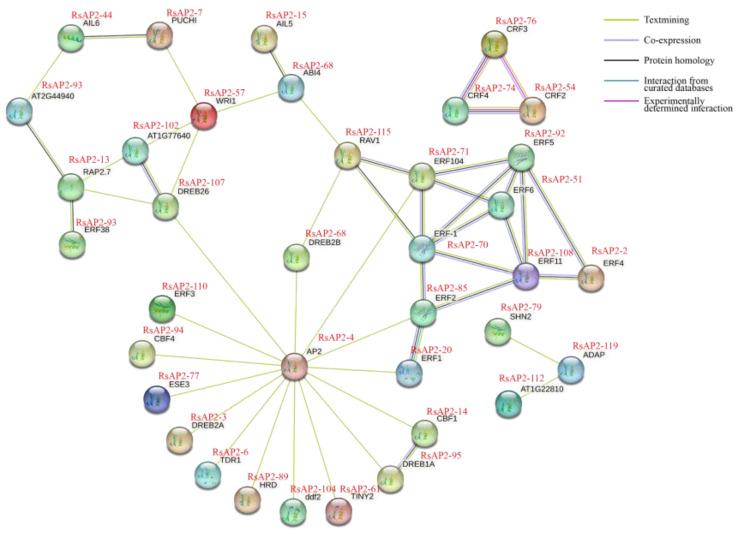
Partial interaction network of RsAP2 protein referring to orthologs in *Arabidopsis*.

The protein interaction network referring to *Arabidopsis* orthologous proteins was constructed to speculate the relationship and synergy of RsAP2 proteins (Figure 8 and Figure S1). According to the result, several RsAP2 genes represented to be core regulators to interact with other proteins. RsAP2-4 had high homology with AP2 protein in *Arabidopsis* and may be upstream from the regulatory network to affect various proteins. The Cytokinin Response Factor 2 (CRF2), CRF3 and CRF4 were determined to have pairwise interaction through the yeast two-hybrid system [31], and the corresponding RsAP2-54, RsAP2-76 and RsAP2-74 may have similar function in response to abiotic stress.

### 2.8. Expression Profiles of RsAP2 Genes under Abiotic Stress

Quantitative Real-Time Fluorescent Polymerase Chain Reaction (qRT-PCR) was conducted to clarify the expression level change in RsAP2 genes with cold, salt and drought stress treatments in closed buds with three biological replicates and two technical replicates. Twenty RsAP2 genes were selected as examples according to the conditions of being confirmed as AP2-like ethylene-responsive transcription factors with annotations of the Pfam, SwissProt and NCBI Non-Redundant (NR) protein databases and had relatively higher FPKM values at the closed bud stage, while their promoters contained cis-acting elements response to abiotic stress and abscisic acid. Some RsAP2 genes showed significant changes in expression levels in response to abiotic stress treatments.

**Figure 9:**
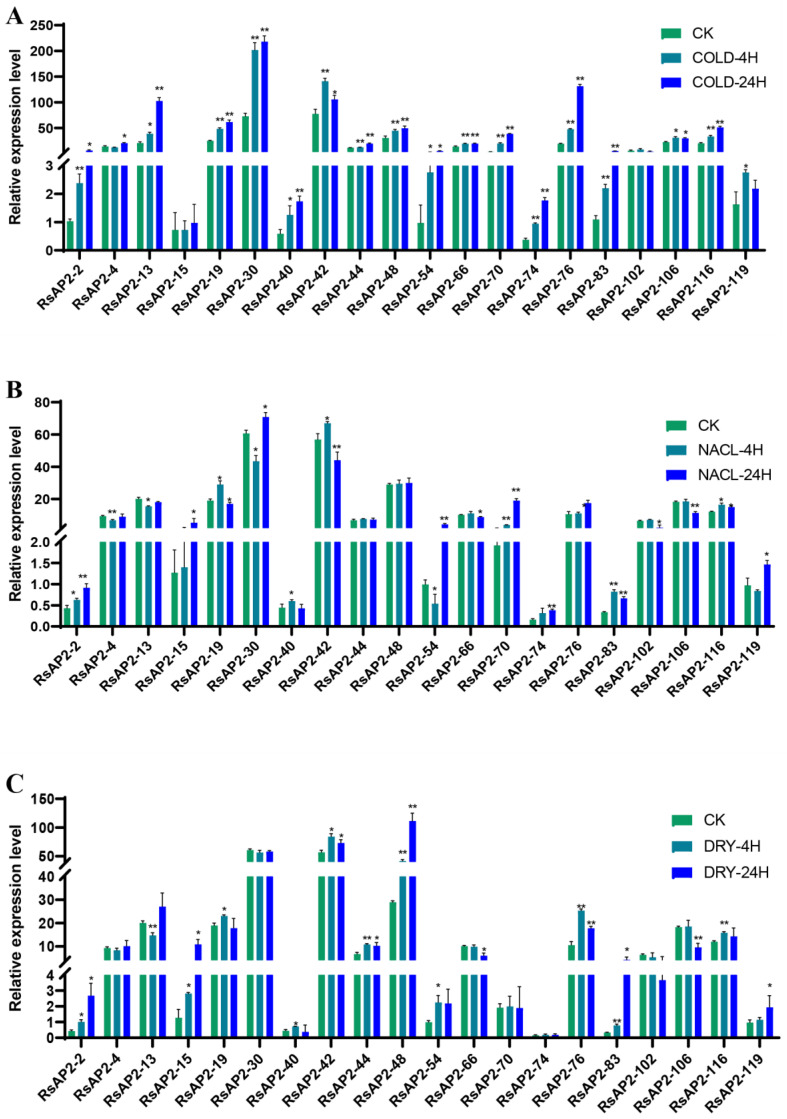
Relative expression level of 20 selected RsAP2 genes in response to cold (**A**), salt (**B**) and drought (**C**) treatments. Actin gene was taken as the internal reference gene. The relative expression levels are presented with mean and standard deviation of qRT-PCR. The significance test is presented with asterisks: * *p* < 0.05, ** *p* < 0.01, Student’s *t*-test.

Overall, most of the RsAP2 genes were sensitive to cold stress, as 16 RsAP2 genes had significantly upregulated relative expression with cold treatment for 4 and 24 h compared to the control treated at room temperature and shielded from light (Figure 9). The peak RsAP2-42, 66, 102, 106 and 119 expression levels occurred at 4 h of cold stress, which may indicate a fast response situation.

Although salt treatment may also cause drought, several RsAP2 genes, such as RsAP2-30 and RsAP2-48, had obvious differences in expression patterns. RsAP2-30 expression had barely any volatility with the drought treatment, but the salt treatment resulted in this gene being significantly downregulated at 4 h but significantly upregulated at 24 h. As for RsAP2-30, the expression was mostly stable with the salt treatment, but the drought treatment resulted in extreme upregulation of the expression at 4 and 24 h. However, the expression patterns were rather similar in RsAP2-2, 15, 19, 40, 66, 106 and 116 with both the salt and drought treatments.

## 3. Discussion

With a large number of family members and diversified functions including abiotic stress resistance, hormone signal transduction and plant metabolite regulation, the AP2/ERF transcription family is important in many plant species. Many species specific AP2/ERF transcripts have been identified with the study of whole-genome sequencing data of many plants such as *Arabidopsis* [32], rice [33] and poplar [34]. Most studies have concentrated on model and crop plants, while only a few studies have detected transcription factor families in ornamental plants. Therefore, we performed a comprehensive and overall study of the AP2/ERF transcript family in *Rhododendron*, which has not been done before.

We identified 120 RsAP2 genes and carried out comprehensive studies on their phylogeny, sequence structure, chromosomal location, collinearity and expression patterns in flower development stages and abiotic stresses. This information can provide a theoretical basis and data support for further research on the functional pathways in stress resistance and flowering physiology of *Rhododendron* and other ornamental plants.

Previous research has reported that the number of AP2/ERF genes was not directly related to genome size, as the numbers of AP2/ERF genes in *Arabidopsis*, rice, poplar and rose were 147, 164, 200 and 135, respectively, while their genome sizes were 125, 430, 485 and 600 Mb, respectively. With 120 AP2/ERF genes identified and a 609 Mb genome size, the findings for *Rhododendron* could support this conclusion.

Phylogenetic trees can be used to study visually the relationships of sequences. In our research, the sequences were not clustered by species firstly but, rather, according to the classification of structure. The AP2 subfamily genes were grouped into an independent cluster, while the DREB and ERF subfamilies had a staggered distribution and formed different subgroups. This may be due to the tandem double AP2 domains, a relatively obvious specific structure.

The conserved motifs are conserved amino acid sequences with various biological functions involved in biological processes such as protein cointeraction, transcriptional activation and nuclear localization [32]. The tandem motif 11-1-8 and motif 3-2 occurred in all of the ERF and DREB subfamily members, while in AP2 subfamily members the tandem motif 6-4-5 was a common structure, suggesting that sequence structures have a close relationship with certain functions.

Some characteristics and commonalities can be found through the analysis of gene structures. There were 79 RsAP2 genes with no introns, which accounted for 65.83% of all the family members, similar to the situation in rose [7]. Interestingly, the RAV subfamily members all had no introns, while the AP2 subfamily members contained more than four introns without exception.

According to many studies, the evolution of genomes and genetic systems cannot occur without gene duplications. Gene families could therefore be the product of segmental duplication and tandem duplication [35]; in these ways, plants can achieve rapid adaption to environmental change [36]. In the present study, the duplication event of RsAP2 genes was analyzed to complete the understanding of the expansion pattern. The distribution of tandem duplications was uneven, with 30 RsAP2 genes clustered into 12 tandem duplication event regions on *Rhododendron* chromosomes 1, 5, 7, 8, 9 and 10, while 50 segmental duplication events involving 71 RsAP2 genes were identified. This indicated that tandem duplication and segmental duplication both contributed greatly to the evolution of *Rhododendron* AP2/ERF genes. Many of the RsAP2 genes associated with tandem duplication events tended to have similar expression patterns in different developing stages of *Rhododendron* flowers, as shown in Figure 7, which is evidence of the close relationship between structure and function.

Cis-acting elements not only determine the tissue-specific expression of genes, but also have close relationship with stress resistance [37]. The cis-acting elements were mainly distributed in the 2 kb upstream sequence and could be analyzed to clarify the mechanisms and potential functions of RsAP2 transcriptional regulation. Among the elements, ABREs had the largest variety of sequence types according to data for four species (Table S2). The ABREs in *Rhododendron* were widely distributed upstream of 108 RsAP2 genes, and 78 of them appeared more than once. The high frequency and wide range of ABRE elements suggest that the function involved in abscisic acid responsiveness to resist abiotic stresses including cold, drought and salt [38], played an important role and was guaranteed in quantity. Another important cis-acting element is mainly involved in low temperature responsiveness [39], which occurred in the promoter of 68 RsAP2 genes. Three kinds of elements with MYB binding sites for MYB TFs, MBS, MBSI and MRE, were detected for their functions of drought-inducibility, flavonoid biosynthetic genes regulation and light responsiveness, respectively. This may imply the relation and interaction of AP2/ERF and MYB transcription factors and the complex regulatory network.

The expression patterns of genes were analyzed to achieve deeper understanding of the AP2/ERF genes in terms of overall trends and individual differences. Transcriptome data were the main data source to reveal the expression level of genes in different states of plant tissue. There is a period of dormancy with special biological significance for the *Rhododendron* flower bud before anthesis. According to the RNA-seq data for *R. simsii*, the RsAP2 genes presented different expression patterns in the five developmental stages of flowers. At the closed buds stage (S1), several RsAP2 genes had peak expression levels, while others had the lowest expression levels. Additionally, combined with the gene duplication events mentioned above, the clustered tandem duplicated genes exhibited high similarities in expression patterns among all five stages. This phenomenon may indicate the important roles of duplicated genes in developmental physiology and stress response because their function may be enhanced through an increase in quantity.

Quantitative RT-PCR can intuitively illustrate the RsAP2 genes in *Rhododendron* flower buds under different abiotic stress treatments. Twenty RsAP2 genes were selected as examples according to conditions, including phylogenetic analysis, annotations, FPKM values, cis-acting elements and protein interaction networks. With cold hardiness being an important breeding goal for researchers around the world, we focused on the gene response to cold stress, which significantly affected most of the selected RsAP2 genes. The accumulation of expression levels was positively correlated with the increase in time treated with cold stress for the overall trend. The functions of RsAP2-30/54/74/76 were supposed to be similar to those of the AT4G27950 gene, with a close phylogenetic relationship. AT4G27950 was regarded as CRF4 and showed transcriptional induction after exposure to cold [40]. Several special cases, such as RsAP2-42/106/119, had their expression peak with 4 h of cold treatment, which may indicate the characteristics of a rapid response. Although the application of higher salt concentrations may cause cellular osmotic water loss resulting in a drought-like effect, some AP2/ERF genes show specific patterns in response to salt treatment compared with mannitol-induced drought treatment, such as RsAP2-70 and RsAP2-48. RsAP2-70 had a close structural relationship with AT1G06160, which was named AtERF15. AtERF15 was reported to be the binding factor of Coupling Element 1 and was a positive regulator of the ABA response [41]. It can be assumed from this that RsAP2-70 was induced with salt stress and regulated by ABA as a downstream response factor. Meanwhile, RsAP2-48 exhibited no significant change in expression level after salt treatment. The function of RsAP2-48 may be similar to that of AT3G20840, the PLETHORA 1 gene in *Arabidopsis*, induced by auxin to control the alteration of stomatal apertures under drought stress [42]. Abiotic stresses, plant growth regulators and TFs can form regulatory networks, and the AP2 gene family has extensive involvement in the process of environmental adaptation. Some of the RsAP2 genes appear to be redundant under abiotic stress and show similar expression patterns; they can be multiple types of insurance in terms of mechanisms or can be functionally cumulative in terms of quantity.

The above analysis was performed to systematically mine the genome-wide AP2 gene family of *Rhododendron*. Precise studies of regulatory systems and molecular mechanisms can provide a theoretical basis for future genetic improvement.

## 4. Materials and Methods

### 4.1. Identification of AP2 Family Members in the Rhododendron Genome

The whole *Rhododendron* protein sequence was generated from the *Rhododendron* genome sequencing data. (The data have been submitted to NCBI genebank under the BioProject number of PRJNA902133). HMM was used as the main method to identify *Rhododendron* AP2 candidates. We downloaded the model file (PF00847) from the Protein Family (Pfam) database (http://pfam.xfam.org/, accessed on 23 June 2022). The first domain search was conducted using the model file with the standard of E-value < 1 × 10^−10^ to achieve a species-specific model in the *Rhododendron* genome protein sequence. Putative protein sequences were achieved with the second search using the species-specific model with the standard of E-value < 1 × 10^−10^. The putative sequences were submitted to the SMART (http://smart.embl-heidelberg.de/, accessed on 28 June 2022), NCBI CDD (https://www.ncbi.nlm.nih.gov/Structure/bwrpsb/bwrpsb.cgi, accessed on 28 June 2022) and Pfam databases to verify the AP2 domain. The resulting sequences with AP2 domains confirmed by all three databases were clarified by removing redundant sequences, and the other sequences were categorized as RsAP2 genes. The RsAP2 genes were sorted according to their chromosome number and their location on the chromosome, and they were named in this order at the same time. The subcellular localization of RsAP2 proteins was predicted with WoLF PSORT (https://wolfpsort.hgc.jp/, accessed on 12 October 2022). The RsAP2 sequences were submitted to the Expert Protein Analysis System (ExPASy, http://web.expasy.org/protparam/, accessed on 28 June 2022) to analyze their molecular weights and theoretical isoelectric points (pI).

### 4.2. Phylogenetic and Classification Analysis

The phylogenetic tree of AP2 genes from *Rhododendron* and *Arabidopsis* was conducted using neighbor-joining method with 1000 bootstrap replications to show their evolutionary relationship with MEGA software (version 5.1) [43]. The phylogenetic tree was further modified with Evolview (https://www.evolgenius.info/evolview/#/treeview, accessed on 29 June 2022) [44]. The *Rhododendron* AP2 genes were classified into five subfamilies, AP2, ERF, DREB, RAV and soloist, depending on the number of AP2 domains and other features in sequence according to previous studies [45]. The ERF and DREB subfamilies were further classified into eleven subgroups according to the topology of phylogenetic tree and classification of the *Arabidopsis* AP2 gene family.

### 4.3. Sequence Analysis

The conserved motifs of RsAP2 genes were identified with MEME (http://meme-suite.org/, accessed on 29 June 2022) [46]. The information of gene structures describing the exon and intron was extract from the gff file of genome sequence data. Visualization of the RsAP2 gene structures was conducted by Gene Structure Display Server (GSDS, http://gsds.gao-lab.org/, accessed on 29 June 2022) and Toolbox for Biologists (TBtools, version 1.098774) [47,48].

### 4.4. Chromosomal Localization and Colinearity Analysis

We used MapChart (version 2.32) to present the localization information for RsAP2 genes on chromosomes [49]. The duplication and expansion of the RsAP2 gene family was analyzed with MCScanX (http://chibba.pgml.uga.edu/mcscan2/MCScanX.zip, accessed on 30 June 2022), and we used CIRCOS (https://circos.ca/, accessed on 5 July 2022) to complete the figure of RsAP2 gene family collinearity [50].

### 4.5. Main Cis-Acting Elements Analysis

We extracted 2 kb upstream sequence of the RsAP2 gene sequences from the genome sequencing data and submitted it to PlantCARE (http://bioinformatics.psb.ugent.be/webtools/plantcare/html/, accessed on 30 June 2022) [51]. Cis-acting elements involved in different kinds of plant growth regulators, response to abiotic stresses, anaerobic induction, meristem expression and flavonoid biosynthetic were identified.

### 4.6. Plant Materials and Abiotic Stress Treatments

The *Rhododendron* cultivar ‘Yanzhi Mi’ grown in the greenhouse at the Jiangsu Academy of Agricultural Sciences (Nanjing, China, 118.881 E, 32.039 N) was used in this study. In November 2022, four-year-old plants with closed buds that were grown in separate pots were grouped according to the treatments to be subjected to different abiotic stresses. For cold stress, the plants were placed in a paper box with a black plastic light-shielding bag. Two groups were placed in a 4 °C artificial cold storage room, while the control group was placed at room temperature. For salt and drought stress, the plants were placed in a greenhouse and treated with 200 mM NaCl or 260 mM mannitol, respectively, while the control group was treated with pure water. Closed buds were collected from every group at 4 and 24 h after treatment, frozen in liquid nitrogen immediately after sampling and then stored in a freezer at −80 °C.

### 4.7. RNA-Seq Data Analysis of RsAP2 Genes

RNA-seq data were obtained from a previous study using *Rhododendron* cultivar ‘Yanzhi Mi’ flowers at 5 different developmental stages, including closed buds (S1), buds showing color at the top but with scales still present (S2), initial flowering stage (S3), full flowering stage (S4) and last flowering stage (S5), to analyze the expression patterns of RsAP2 genes during the flower development process (PRJNA700000) [52]. We created a local BLAST database with the Unigenes in the RNA-seq data and processed the tblastn search program with the RsAP2 protein sequence using BioEdit software (version 7.2.5). The corresponding relations were confirmed for the match of query–subject pairs with the lowest E-value, less than 1 × 10^−50^. We used TBtools (version 1.098774) to construct the heatmap with the FPKM values transformed by log2. The RsAP2 protein interaction network was constructed by STRING (https://cn.string-db.org/, accessed on 4 January 2023) with the reference organism of *A. thaliana.*

### 4.8. Total RNA Extractions and Expression Analysis of RsAP2 Genes

Total RNA extraction was conducted with a FastPure Universal Plant Total RNA Isolation Kit (Vazyme, Nanjing, China). The cDNA reverse-transcription process was conducted with HiScript III RT SuperMix for qPCR (Vazyme, Nanjing, China). The primers were designed with Primer Premier software (version 5.0) and synthesized by General Biol Company (Anhui, China). The quantitative real-time PCR process was conducted with ChamQ Universal SYBR qPCR Master Mix (Vazyme, Nanjing, China) on the platform of ROCHE (LightCycler^®^ 480 II, Swiss). The RT-qPCR reaction was conducted in a 96-well plate containing 10.0 μL SYBR Mix, 0.4 μL 10 μM solution of primer, respectively, 0.18 μL undiluted cDNA and 9.02 μL double distilled H2O. The qPCR program was conducted in 3 steps: pre-denaturation stage had 1 cycle of 95 °C for 30 s; recirculation reaction stage had 40 cycles of 95 °C for 10 s and 60 °C for 30 s; melting curve had 1 cycle of 95 °C for 15 s, 60 °C for 60 s and 95 °C for 15 s, in order. Three repeats were performed for each sample. Housekeeping gene *Actin* was taken as the internal reference gene [53]. Relative expression levels of the genes were calculated using the 2^-ΔΔCT^ formula [54].

## 5. Conclusions

This research focused on the analysis of *Rhododendron* AP2/ERF gene family. A total of 120 AP2/ERF genes were identified and their structural features described. The genes were classified into five subfamilies according to their domain and sequence structural characteristics. The phylogenetic comparison of AP2/ERF genes from *Arabidopsis* provided a valuable reference for the evolutionary characteristics of *Rhododendron* AP2/ERF genes. Many of the RsAP2 genes may participate in the development of *Rhododendron* and could be response factors to abiotic stress, such as cold, salt and drought. The expression patterns in different developmental stages and under different abiotic stress treatments indicated the different functions of RsAP2 members. This research generated relatively comprehensive information on the RsAP2 gene family and provides a theoretical basis for future further validation of RsAP2 gene function and protein interaction.

## Data Availability

Data are contained within the article and Supplementary Materials.

## Supplementary Materials

The following supporting information can be downloaded at: https://www.mdpi.com/article/10.3390/plants12050994/s1, Figure S1: Interaction network of RsAP2 protein referring to orthologs in *Arabidopsis*. Table S1: Features of RsAP2. Table S2: Cis-acting elements of RsAP2 promoters.
