# Supplementary material for: Genome-Wide Analysis of the Rhododendron AP2/ERF Gene Family: Identification and Expression Profiles in Response to Cold, Salt and Drought Stress
Source: Plants (Basel). 2023 Feb 22;12(5):994. doi: 10.3390/plants12050994 (PMC10005251; doi:10.3390/plants12050994)
Supplement: Supplementary file 1 [file plants-12-00994-s001.zip › Table S2 Cis-acting elements of RsAP2 promoters .pdf]

Table S2. Cis-acting elements of RsAP2 promoters

Element name	Sequence	Species	Putative function
TGA-element	AACGAC	Brassica oleracea	auxin-responsive
AuxRE	TGTCTCAATAAG	Glycine max	auxin-responsive
TGA-box	TGACGTAA	Glycine max	auxin-responsive
AuxRR-core	GGTCCAT	Nicotiana tabacum	auxin-responsive
ABRE	AACCCGG	Arabidopsis thaliana	abscisic acid-responsive
ABRE	ACGTG	Arabidopsis thaliana	abscisic acid-responsive
ABRE	CACGTG	Arabidopsis thaliana	abscisic acid-responsive
ABRE	TACGGTC	Arabidopsis thaliana	abscisic acid-responsive
ABRE	CGCACGTGTC	Hordeum vulgare	abscisic acid-responsive
ABRE	CGTACGTGCA	Hordeum vulgare	abscisic acid-responsive
ABRE	GCAACGTGTC	Hordeum vulgare	abscisic acid-responsive
ABRE	GACACGTACGT	Oryza sativa	abscisic acid-responsive
ABRE	GCCGCGTGGC	Oryza sativa	abscisic acid-responsive
ABRE	TACGTGTC	Oryza sativa	abscisic acid-responsive
ABRE	GACACGTGGC	Triticum aestivum	abscisic acid-responsive
GARE-motif	TCTGTTG	Brassica oleracea	gibberellin-responsive
P-box	CCTTTTG	Oryza sativa	gibberellin-responsive
TATC-box	TATCCCA	Oryza sativa	gibberellin-responsive
TCA-element	TCAGAAGAGG	Brassica oleracea	salicylic acid-responsive
TCA-element	CCATCTTTTT	Nicotiana tabacum	salicylic acid-responsive
SARE	TTCGACCATCTT	Nicotiana tabacum	salicylic acid-responsive
CGTCA-motif	CGTCA	Hordeum vulgare	MeJA-responsive
TGACG-motif	TGACG	Hordeum vulgare	MeJA-responsive
MRE	AACCTAA	Petroselinum crispum	Light-responsive
LTR	CCGAAA	Hordeum vulgare	Low temperature-responsive
MBS	CAACTG	Arabidopsis thaliana	drought-inducibility
ARE	AAACCA	Zea mays	anaerobic induction
CAT-box	GCCACT	Arabidopsis thaliana	meristem expression
MBSI	aaaAaaC(G/C)GTTA	Petunia hybrida	flavonoid biosynthetic
MBSI	TTTTTACGGTTA	Petunia hybrida	flavonoid biosynthetic
CCAAT-box	CAACGG	Hordeum vulgare	MYBHv1 binding site
