# Supplementary figures and images for: Genome-Wide Analysis of the Rhododendron AP2/ERF Gene Family: Identification and Expression Profiles in Response to Cold, Salt and Drought Stress
Source: Plants (Basel). 2023 Feb 22;12(5):994. doi: 10.3390/plants12050994 (PMC10005251; doi:10.3390/plants12050994)

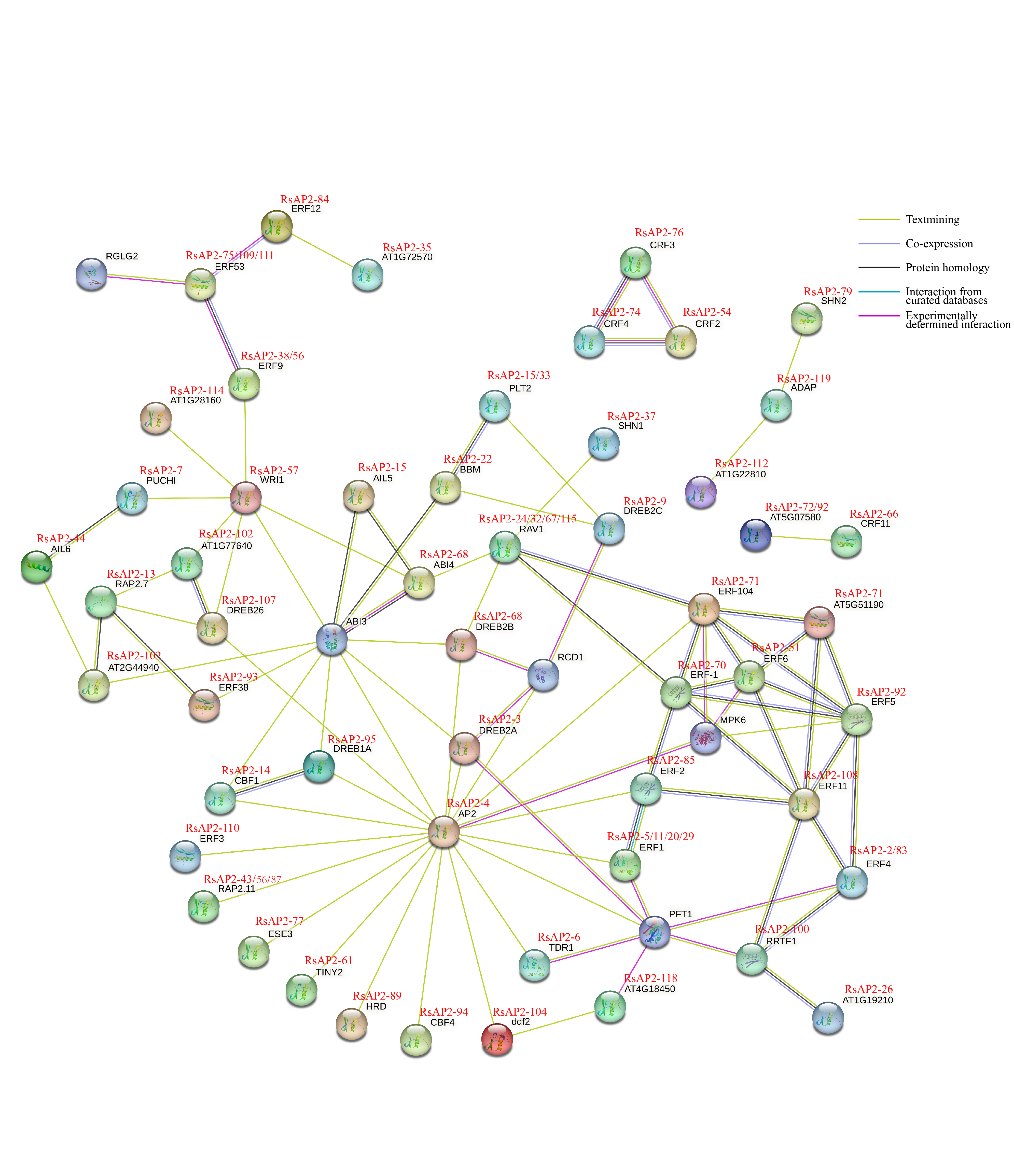

Supplement: Supplementary file 1 [file plants-12-00994-s001.zip › Figure S1 Interaction network of RsAP2 protein referring to orthologs in Arabidopsis..jpg]
